# Genetic Diversity and Molecular Evolution of Porcine Epidemic Diarrhea Virus in Chongqing, China (2022–2024)

**DOI:** 10.3390/ani16132033

**Published:** 2026-07-02

**Authors:** Qianlin Chen, Shaomei Li, Wenjie Ma, Yassein M. Ibrahim, Jie Luo, Yuandi Yu, Lizhi Fu, Qingyong Guo

**Affiliations:** 1 College of Veterinary Medicine, Xinjiang Agricultural University, Urumqi 830052, China; qlchenjj@163.com; 2 Chongqing Academy of Animal Science, Chongqing 402460, China; shaomeili123@163.com (S.L.); roger0601@163.com (J.L.); yuyuandi@126.com (Y.Y.); 3 National Center of Technology Innovation for Pigs, Chongqing 402460, China; qd1992mwj@163.com (W.M.); yassin8322@gmail.com (Y.M.I.); 4 Rongchang, Animal Disease, Observation and Research Station, Ministry of Agriculture and Rural Affairs, Chongqing 402460, China; 5 Chongqing Engineering Technology Research Center for Veterinary Biological Products, Chongqing 402460, China; 6 Faculty of Veterinary Science, University of Nyala, Nyala 155, Sudan

**Keywords:** porcine epidemic diarrhea virus, epidemiology, S gene, molecular phylogeny, genetic variation

## Abstract

Porcine epidemic diarrhea virus (PEDV) continues to undergo genetic evolution and remains a major etiological agent of enteric disease in swine, causing significant economic losses worldwide. This study investigated the molecular epidemiology and genetic characteristics of PEDV circulating in Chongqing, China, between 2022 and 2024. A total of 296 diarrheic piglet samples collected from nine regions were screened using RT-qPCR, of which 48.31% (143/296) tested positive for PEDV. A subset of positive samples was subjected to S gene amplification and sequencing, yielding 15 complete sequences. Phylogenetic analysis revealed that all sequenced strains clustered within the G2c lineage and showed high nucleotide similarity (93.37–94.09%) to the classical CV777 strain. Recombination analysis indicated potential recombination events among field strains involving S-INDEL and G2b-like parental lineages, although these findings are based on a limited number of sequences. Sequence analysis identified multiple amino acid substitutions within the COE antigenic region, while other neutralizing epitopes (SS2, SS6, and 2C10) remained largely conserved. In addition, variation in predicted N-glycosylation sites was observed among some strains. Structural modelling suggested that these changes may influence spike protein conformation and antigenic properties; however, these interpretations are based on in silico analysis and require experimental validation. Overall, the findings indicate ongoing genetic evolution of PEDV in Chongqing and suggest circulation of G2c-associated variants in diarrheic piglets. However, given the limited and non-random nature of sequencing, these results may not fully represent the broader viral population. Continued large-scale molecular surveillance and functional studies are needed to better understand PEDV evolution and to support the development of improved control strategies and vaccines.

## Simple Summary

Porcine epidemic diarrhea virus (PEDV) continues to evolve rapidly, while existing vaccines provide limited protection, posing major threats to global swine production. RT-qPCR screening of 296 clinical samples from diarrheic piglets collected in Chongqing between 2022 and 2024 revealed a PEDV positivity rate of 48.31%. Phylogenetic analysis showed that all local strains belonged to the G2c sublineage and exhibited conserved recombination patterns, distinct COE amino acid substitutions, and variable N-glycosylation sites. These genetic changes may influence viral pathogenicity, antigenicity, and immune evasion capacity, contributing to the continued predominance of the G2c sublineage. This study enhances current PEDV epidemiological data in Southwest China and supports targeted vaccine development.

## 1. Introduction

Neonatal suckling piglets are highly susceptible to porcine epidemic diarrhea virus (PEDV), which causes acute enteric disease characterized by severe diarrhea, dehydration, and high mortality, with fatality rates approaching 100% in piglets younger than 7 days [1,2]. Porcine epidemic diarrhea (PED) was first reported in the UK in 1971, and the prototype PEDV strain CV777 was subsequently isolated and characterized in Belgium in 1978 [3]. After the first emergence in China in 1984, PED remained sporadic for several decades [4]. However, the emergence of a highly virulent G2 subgroup in China in October 2010 triggered a nationwide epidemic [5]. In the following years, analogous outbreaks were reported across numerous countries and regions, causing devastating suckling piglet mortalities and substantial economic losses to the global swine sector [6,7,8]. PEDV is taxonomically classified under the order Nidovirales, family Coronaviridae, genus Alphacoronavirus. It is an enveloped, single-stranded positive-sense RNA virus with an approximately 28 kb genome, containing seven open reading frames (ORFs) arranged as follows: 5′UTR-ORF1a-ORF1b-S-ORF3-E-M-N-3′UTR. ORF1a encodes replicase polyprotein 1a (pp1a). A programmed −1 ribosomal frameshift enables translation read-through from ORF1a into ORF1b to generate full-length polyprotein pp1ab. This precursor is subsequently cleaved by viral proteases into non-structural proteins nsp1–nsp16. The remaining ORFs sequentially encode spike (S), accessory ORF3, envelope (E), membrane (M), and nucleocapsid (N) proteins [7]. The S, E, and M proteins form the viral envelope, whereas the N protein associates with the genomic RNA to form the nucleocapsid [1].

The S protein, encoded by the S gene, is a type I transmembrane trimeric glycoprotein located on the virion surface. It acts as the key functional molecule mediating host receptor recognition, viral attachment, membrane fusion, and cellular entry [9]. Notably, the S gene is highly variable and frequently undergoes recombination, substitution, insertion, and deletion. These genetic changes significantly influence PEDV pathogenicity, transmissibility, and evolutionary dynamics, making the S gene a key target for molecular epidemiological and phylogenetic analyses [10,11]. Based on whole-genome and S gene homology comparisons, PEDV strains are categorized into classical G1 and variant G2 clades [12]. The G1 clade includes G1a (prototype CV777-like strains) and G1b (early classical isolates such as SD-M). The G2 clade comprises three lineages: G2a (early U.S. field strains such as AH2012), G2b (Chinese epidemic strains from 2010–2020 including AJ1102 and LC), and G2c (recent Chinese variants such as SD2021 and TJbc2023) [13]. Nearly all PED outbreaks reported since 2010 have been linked to G2 variants [14]. This epidemiological shift is likely driven by sustained immune selection pressure resulting from widespread vaccination with strains belonging to G1a (CV777), G1b (ZJ08), and G2b (AJ1102) sublineages. Such pressure accelerates viral evolution, particularly within the S gene, leading to the emergence of novel epidemic strains with altered virulence and transmissibility [15,16]. Although commercial vaccines are widely used in Chinese swine herds, field strains persist under immune selection pressure through two adaptive traits: high sialic acid binding affinity and immune escape, indicating that current vaccines provide insufficient cross-protection against such strains [17]. Therefore, continuous epidemiological surveillance and genetic characterization of circulating PEDV variants remain essential for optimizing prevention and control strategies.

To characterize the prevailing epidemiological profile and genetic variation in PEDV in Southwest China, clinical samples from diarrheic piglets covering nine districts and counties of Chongqing were tested via real-time reverse transcription PCR (RT-qPCR). Representative PEDV-positive field samples were further subjected to amplification and sequencing for comprehensive genetic and polymorphism analysis. Our results provide valuable data for long-term surveillance regarding the epidemiological trends and evolutionary dynamics of endemic PEDV strains in Chongqing. Furthermore, these findings provide important molecular baseline evidence to support optimizing integrated PED prevention and control strategies, as well as developing more effective strain-specific vaccines.

## 2. Materials and Methods

### 2.1. Clinical Samples

Sample collection was conducted from September 2022 to July 2024. A total of 296 clinical specimens, including intestinal tissues and fecal samples, were collected from PED-suspected piglets on 18 commercial swine farms across nine districts and counties of Chongqing (Supplementary Table S1). All sampling was completed by field veterinarians with prior approval from farm managers. Specimens were temporarily stored in portable refrigerated biosafety containers, transported to the laboratory via cold chain, and stored at −80 °C prior to testing.

### 2.2. Nucleic Acid Extraction

The samples stored at −80 °C were thawed on ice prior to RNA extraction. Briefly, 0.2 g of each fecal specimen was transferred to RNase-free homogenization tubes and homogenized in prechilled PBS at 60 Hz for 1 min using a tissue homogenizer (Jingxin Co., Ltd., Shanghai, China). Intestinal tissues were rinsed with the same prechilled PBS; approximately 0.1 g of mucosal tissue was homogenized twice (60 Hz, 1 min each), with a 2 min incubation at 4 °C between cycles. All homogenates were centrifuged at 15,294× *g* for 10 min at 4 °C; clarified supernatants were harvested for viral RNA extraction. Total viral RNA was extracted following the manufacturer’s instructions for the Tianlong RNA extraction kit (Xi’an Tianlong Science and Technology Co., Ltd., Xi’an, China) using its matching automated nucleic acid extraction system. RNA concentration and purity were assessed with the NanoDrop ONE ultramicro spectrophotometer (Thermo Fisher Scientific, Waltham, MA, USA). Qualified RNA was aliquoted and stored at −80 °C for subsequent testing.

### 2.3. RT-qPCR Detection

RT-qPCR was performed using a validated assay established in our laboratory [18]. Target-specific primers and probes were designed based on the conserved sequence of the PEDV M gene (sequences listed in Supplementary Table S2). The amplification was carried out on a CFX96^TM^ Real-Time PCR Detection System (Bio-Rad Laboratories, Hercules, CA, USA) with a one-step RT-qPCR kit (Takara Bio Inc., Kusatsu, Shiga, Japan). The 20 μL reaction system contained 1 μL template RNA, 0.5 μM forward and reverse primers, and 0.3 μM probe. Thermal cycling conditions were as follows: reverse transcription at 52 °C for 5 min, initial denaturation at 95 °C for 10 s, and 40 cycles of denaturation at 95 °C for 5 s, annealing and extension at 61 °C for 30 s. Fluorescence signals were acquired after each extension cycle. Recombinant pMD19T-M carrying the PEDV M fragment was used as the positive control. Negative and blank controls were included in each run to verify assay reliability. Assays were valid if positive control Ct values ranged from 15 to 23, while negative and blank controls exhibited no amplification (Ct > 38 or undetected). Samples with Ct < 35 were PEDV-positive. Representative PEDV-positive intestinal tissue samples from each region were subjected to S gene amplification, cloning and sequencing to characterize circulating PEDV strains.

### 2.4. S Gene Cloning and Sequencing

Partial amplification primers targeting the PEDV S gene were designed from conserved regions of its full-length sequence, including two pairs of specific primers (PEDV-S1F/S1R and PEDV-S2F/S2R; primer sequences are provided in Supplementary Table S2). Total RNA extracted from positive specimens was reverse-transcribed into first-strand cDNA with the HiScript IV 1st Strand cDNA Synthesis Kit (Vazyme Biotech Co., Ltd., Nanjing, China). The target gene fragments were subsequently amplified via high-fidelity PCR using 2 × Phanta Max Master Mix (Dye Plus, Cat. No. P525, Vazyme) following the manufacturer’s instructions. Each 20 μL PCR system contained 10 μL of the above master mix, 0.5 μM forward and reverse primers, and 2 μL cDNA template. The optimized thermal cycling conditions consisted of an initial denaturation at 95 °C for 5 min, followed by 35 cycles of denaturation at 95 °C for 15 s, annealing at 60 °C for 15 s, and extension at 72 °C for 2 min 30 s, with a final extension at 72 °C for 5 min. PCR products were first analyzed via 1% agarose gel electrophoresis. DNA bands of the expected sizes were excised and purified with the EasyPure^®^ Quick Gel Extraction Kit (Cat. No. EG101, TransGen Biotech Co., Ltd., Beijing, China). Purified amplicons were ligated into cloning vectors and transformed into competent cells using the pEASY^®^-Blunt Cloning Kit (Cat. No. CB101, TransGen). Positive recombinant colonies were screened by PCR using M13 universal primers. For each valid sample, two confirmed positive clones were selected and sent to Shanghai Sangon Biotech Co., Ltd. (Shanghai, China) for bidirectional sequencing.

### 2.5. Phylogenetic and Homology Analysis of the S Gene

Raw sequencing data of S genes amplified from Chongqing-derived PEDV field specimens were assembled and edited using SnapGene software (v8.0), yielding full-length sequences. A total of 88 publicly available PEDV reference sequences were retrieved from the NCBI GenBank database (detailed information provided in Supplementary Table S3). Multiple sequence alignment of the obtained full-length sequences and reference strains was performed using MEGA (v11.0) with the MUSCLE algorithm. Based on the aligned datasets, a neighbor-joining (NJ) phylogenetic tree was constructed with 1000 bootstrap replicates to evaluate branch reliability, then annotated and visualized via the Interactive Tree Of Life (iTOL, https://itol.embl.de/; accessed on 3 May 2026) online platform. Sequence homology analysis of the S gene was also conducted in the same software to evaluate the genetic relatedness between local field and reference sequences from different PEDV subclusters. Nucleotide and amino acid homology matrices were calculated using the formula [(1-p-distance) × 100%]. The resulting matrix data were imported into RStudio software (v2026.04.0) for the construction of sequence homology heatmaps. A continuous gradient color scale was applied, with variations in color intensity representing differences in sequence homology among strains. Additionally, amino acid sequence alignment was performed to compare the major antigenic epitope regions of the S protein, including COE, SS2, SS6, and 2C10, to characterize mutation patterns and conservation profiles of these key functional domains.

### 2.6. Recombination Analysis

Potential recombination events in the S gene of Chongqing PEDV strains were analyzed. All sequenced S gene fragments, together with representative standard reference sequences, were analyzed for recombination using RDP4 software (v4.39). Seven recombination detection algorithms, namely RDP, GENECONV, Chimaera, MaxChi, BootScan, SiScan, and 3Seq, were applied simultaneously using default parameters to ensure comprehensive analysis. Reliable recombination events were considered credible only when at least six of the seven methods identified consistent signals, with a statistical significance threshold of *p* < 1.0 × 10^−6^. To further validate the detected recombination events and minimize false-positive results, all candidate recombination signals were subsequently confirmed using SimPlot software (v3.5.1).

### 2.7. Analysis of Potential N-Glycosylation Sites on the S Protein

To characterize the distribution of putative N-glycosylation sites on the S protein of endemic PEDV, full-length nucleotide sequences of representative field specimens were translated into corresponding amino acid sequences. Potential N-glycosylation sites were predicted using the online NetNGlyc-1.0 platform (https://services.healthtech.dtu.dk/services/NetNGlyc-1.0/; accessed on 7 May 2026) with default parameters. Based on the screening criteria of a prediction score > 0.5 and neural network agreement of at least 7/9, the spatial distribution of predicted N-glycosylation sites was systematically compared between local PEDV variants and representative reference strains from different genetic sublineages.

### 2.8. Spatial Analysis of Mutant Amino Acids in the COE Antigenic Domain

To analyze substitutions in the COE domain of Chongqing PEDV sequences and their impacts on protein structure and function, full-length nucleotide sequences were translated into amino acid sequences. The crystal structure of the prototype CV777 strain (PDB ID: 6VV5) was used as the structural template. ChimeraX (v1.11.1) was used to map and visualize the COE domain mutation sites onto the tertiary structure of the PEDV S protein. Structural superposition was further performed to identify conformational differences induced by site-specific mutations among circulating strains and to assess their potential functional implications.

## 3. Results

### 3.1. RT-qPCR Test Results

**Figure 1:**
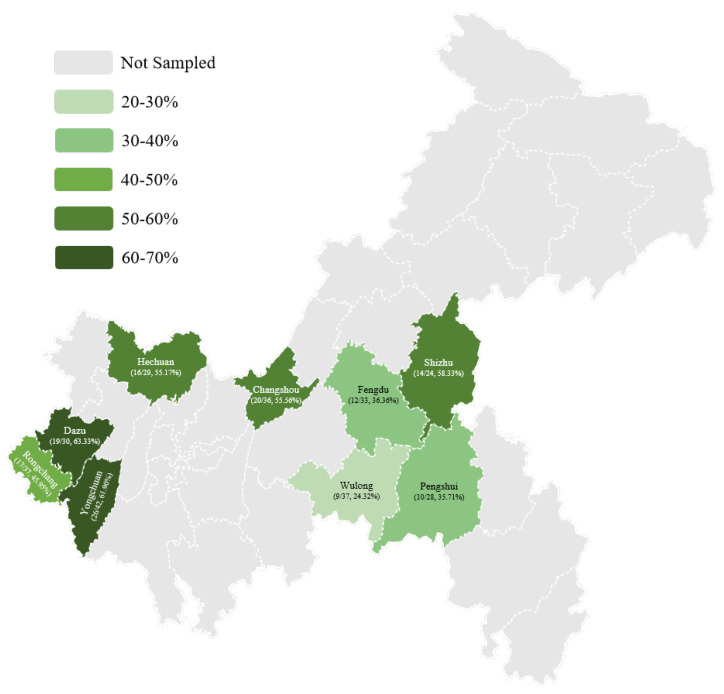
Geographic distribution of PEDV-positive samples across Chongqing. Gray areas denote unsampled districts. Regional PEDV positivity rates were calculated as (positive samples/total samples) × 100%.

From 2022 to 2024, a total of 296 clinical samples were collected from diarrheic piglets across nine districts and counties of Chongqing to investigate the regional epidemiological distribution of PEDV. All samples were tested for PEDV using a laboratory-established RT-qPCR assay. Molecular screening revealed an overall PEDV-positive rate of 48.31% (143/296), and the positive samples were detected in all surveyed regions, with a farm-level positivity rate of 88.89% (16/18). Marked geographic variation in PEDV prevalence was observed among the different sampling regions, with regional positivity rates ranging from 24.32% to 63.33%. Within the central urban zones, Dazu District exhibited the highest positivity rate (63.33%, 19/30), followed closely by Yongchuan District (61.90%, 26/42). Hechuan and Changshou Districts showed comparable infection levels, whereas Rongchang District showed the lowest positivity rate (45.95%, 17/37) within this region. In the southeastern Wuling mountainous area, Shizhu County had the highest positivity rate (58.33%, 14/24), followed by Pengshui County (35.71%, 10/28), while Wulong District had the lowest rate (24.32%, 9/37). In the northeastern Three Gorges Reservoir region, Fengdu County showed a positivity rate of 36.36% (12/33) (Figure 1; Supplementary Table S1).

### 3.2. Results of S Gene-Based Phylogenetic Analysis

**Figure 2:**
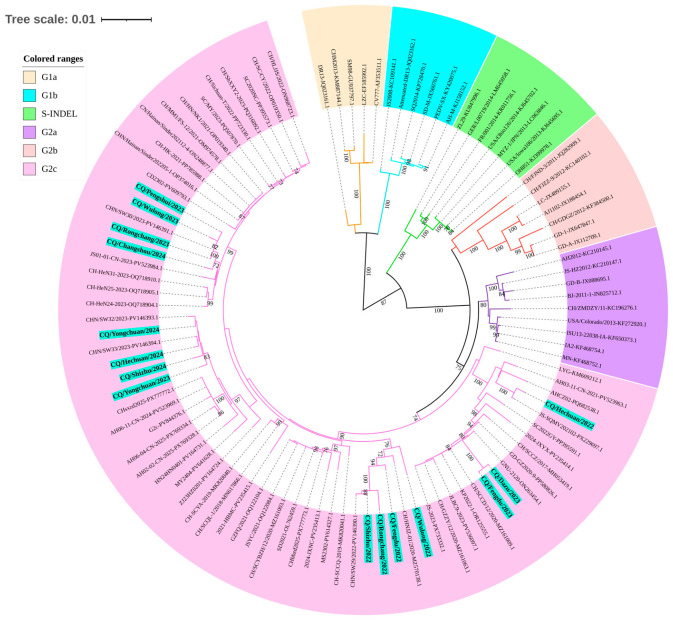
Phylogenetic tree of PEDV generated from full-length S gene sequences via the neighbor-joining method in MEGA (v11.0). 1000 bootstrap replicates were used to test the robustness of the tree topology. Only bootstrap support values over 70 were displayed at each node. All strain identifiers and corresponding GenBank accessions were marked at the tips of branches. Chongqing endemic strains from this study are highlighted in bold blue shading.

To characterize the evolutionary features and dominant genotype distribution of endemic PEDV in Chongqing, 18 PEDV-positive intestinal tissue samples with relatively low Ct values were screened from nine local regions for subsequent molecular analysis. Target fragment amplification was performed via RT-PCR using S gene-specific primer pairs and high-fidelity DNA polymerase, and target-length amplicons were obtained from all samples. Purified PCR products were inserted into the pEASY^®^-Blunt cloning vector for Sanger sequencing. Raw sequencing reads were assembled using SnapGene (v8.0). Full-length S gene sequences were successfully obtained from all 18 samples; among these sequences, 15 were unique, and the remaining three shared 100% nucleotide identity with three of the 15 unique sequences. After deduplication, 15 unique full-length S sequences with four distinct lengths were retained for further analysis: one of 4149 bp, one of 4152 bp, four of 4158 bp, and nine of 4161 bp. All sequences were deposited in NCBI GenBank, and their accession numbers are provided in Supplementary Table S3. For phylogenetic analysis, these sequences were aligned against 88 representative PEDV reference strains retrieved from NCBI. As shown in Figure 2, all 15 Chongqing sequences clustered within the G2c sublineage, indicating their close genetic relationship with recently circulating PEDV strains.

### 3.3. Results of S Gene Identity Analysis

**Figure 3:**
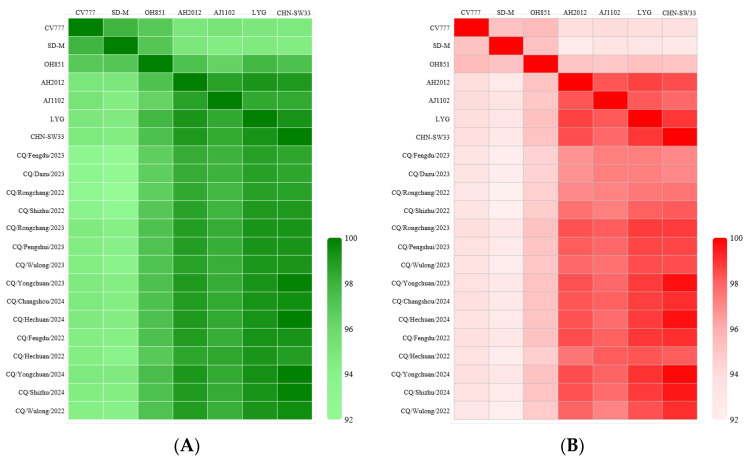
Identity heatmap of PEDV S gene nucleotide and deduced amino acid sequences. (**A**) Green blocks show nucleotide identity between sequenced and reference strains. (**B**) Red blocks show amino acid identity.

Nucleotide identity analysis of full-length S genes revealed pairwise identities ranging from 97.45% to 99.95% among the 15 Chongqing field strains. Compared with representative strains of distinct PEDV sublineages, our sequences shared 93.37–94.09% nucleotide identity with prototype G1a strain CV777 (AF353511.1), 93.13–93.97% with G1b strain SD-M (JX560761.1), and 95.44–96.33% with the S-INDEL variant OH851 (KJ399978.1). For G2 reference sequences, the local strains shared 97.35–98.51% identity with G2a AH2012 (KC210145.1), 96.79–97.67% with G2b strain AJ1102 (JX188454.1), and 98.07–98.92% with the representative G2c strain LYG (KM609212.1). The pairwise identity with the Chongqing G2c strain CHN/SW33/2023 (PV146394.1) reached 97.74–99.90%. The corresponding amino acid sequences were translated for comparative analysis. The 15 local strains exhibited 96.60–99.86% amino acid identity. Their identity to CV777, SD-M, OH851, AH2012, AJ1102, LYG, and CHN/SW33/2023 was 92.88–93.56%, 92.08–92.83%, 94.42–95.87%, 96.97–98.41%, 97.25–98.05%, 97.33–98.92% and 97.18–99.78%, respectively. Nucleotide and amino acid identities between sequenced and reference strains are visualized in the heatmap (Figure 3; Supplementary Tables S4 and S5).

### 3.4. Comparison of Major Antigenic Epitopes in the S Protein

**Figure 4:**
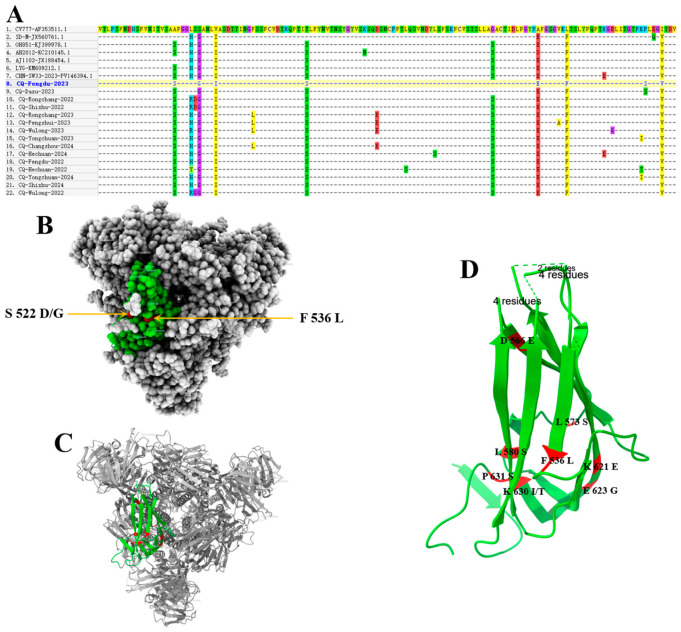
Amino acid variants and their spatial distribution within the COE domain of the S protein. (**A**) COE sequence alignment of local field strains and seven reference strains. (**B**) Surface rendering of the full S trimer; COE (green), variant residues (red). (**C**) Ribbon diagram of the S trimer highlighting variant residues. (**D**) 3D structure of the A-chain COE showing variant spatial distribution.

Four major antigenic epitopes of the PEDV S protein, including COE (499–638 aa), SS2 (748–755 aa), SS6 (764–771 aa), and 2C10 (1368–1374 aa) [19], were selected for comparative epitope analysis. Variations in these functional domains were analyzed between the Chongqing endemic and seven representative reference strains: CV777, SD-M, OH851, AH2012, AJ1102, LYG, and CHN/SW33/2023. As shown in Figure 4A, ten amino acid variants were detected in the COE domain of these strains, including S522D/G, F536L, D566E, L573S, L580S, V610A, K621E, E623G, K630I/T, and P631S. Residue 521 displayed polymorphism: two of these strains harbored leucine consistent with CV777, eight carried histidine matching other sublineage reference sequences, four carried arginine, and one carried tyrosine. Residue 594 harbored glycine in two strains, an amino acid characteristic of G1 lineage strains. SS2, SS6 and 2C10 epitope regions remained highly conserved, with no insertions, deletions or amino acid variants observed. ChimeraX (v1.11.1) was used to map the spatial distribution of prevalent COE variants on the CV777 S protein crystal structure. The variants display distinct spatial locations: D566E, L573S and L580S are embedded in the internal core β-sheets; F536L, K621E and E623G reside on solvent-exposed flexible loops protruding from these sheets; K630I/T and P631S localize to the flexible C-terminus of this domain (Figure 4B–D).

### 3.5. Recombination Analysis of the S Gene

**Figure 5:**
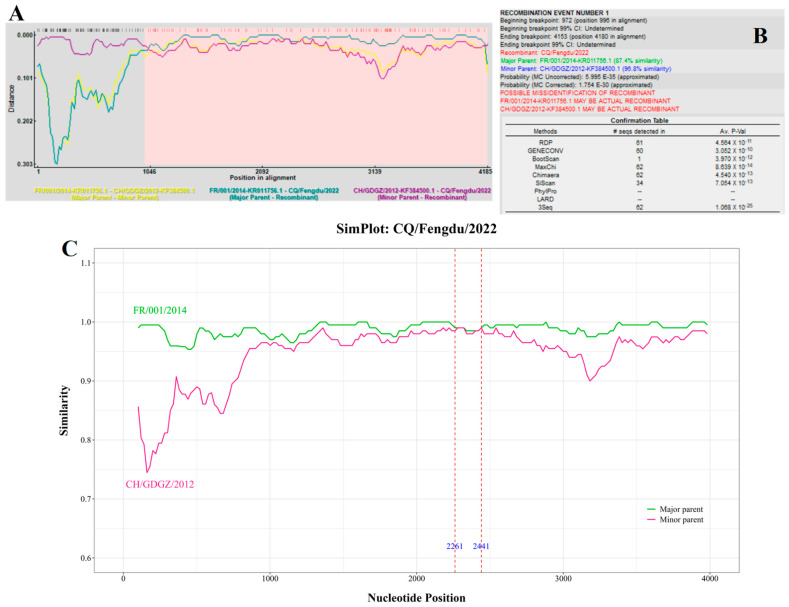
Recombination profiles of the S gene from the representative strain CQ/Fengdu/2022. (**A**) RDP4 recombination prediction. Yellow curve: similarity between two parents; green curve: similarity between major parent and CQ/Fengdu/2022; purple curve: similarity between minor parent and CQ/Fengdu/2022. Pink shading indicates the predicted 1293–4158 nt recombinant region. (**B**) Seven-algorithm recombination validation. (**C**) SimPlot (v3.5.1) breakpoint verification. Breakpoints are located at 2261 nt and 2441 nt.

Recombination events in the S gene of newly obtained PEDV strains were detected using RDP4 (v4.39). Of the 15 field strains, 11 met the stringent statistical threshold of *p* < 10^−6^ across all seven detection algorithms. These recombinants exhibited highly consistent recombination patterns and similar breakpoint positions. Sequence alignment revealed that these recombinants shared the same major parent FR/001/2014 (KR011756.1) and minor parent CH/GDGZ/2012 (KF384500.1). Three distinct recombination intervals were identified among these strains: 996–4180 nt (6 strains), 1112–4180 nt (2 strains), and 1293–4180 nt (3 strains), with most internal sequences matching the major parent FR/001/2014. The remaining four strains showed weak recombination signals but failed to meet the rigorous screening criteria and thus were excluded from further recombination analysis. To validate the parental origins and precisely define recombination breakpoints, SimPlot (v3.5.1) was subsequently employed. As illustrated in Figure 5A, RDP4 predicted a 996–4180 nt recombination interval for the representative strain CQ/Fengdu/2022, and the corrected *p*-value from the seven-algorithm analysis was 1.754 × 10^−30^ (Figure 5B), confirming intragenic recombination. SimPlot further pinpointed breakpoints at 2261 nt and 2441 nt within this broad segment (Figure 5C). Specifically, the 1–2261 nt and 2441–4158 nt regions exhibited higher similarity to the major parent FR/001/2014, while the 2261–2441 nt segment constituted the crossover interval with nearly equal similarity to both parents.

### 3.6. Prediction of Potential N-Glycosylation Sites on the S Protein

N-glycosylation sites on the S protein of newly sequenced field strains were analyzed (Table 1). Nine N-glycosylation sites were highly conserved among representative strains from distinct subtypes: 213 NVTS, 422 NFTG, 511 NITV, 553 NVTN, 685 NVTS, 740 NCTE, 778 NISI, 1246 NKTL, and 1275 NLTG. A total of 18–20 putative sites were identified across 15 Chongqing field strains. Two field strains (CQ/Fengdu/2023 and CQ/Dazu/2023) harbored two novel sites (300 NKTI, 863 NISS) and lacked the canonical 341 NLSF site; their mutational profiles aligned with the minor parent strain CH/GDGZ/2012. In addition, a novel 1196 NRTA site was detected in CQ/Wulong/2023 and CQ/Yongchuan/2023, whereas the 787 NFSV site was only found in the dominant strain CHN/SW33/2023 and three other field strains. Notably, these four novel glycosylation sites (300 NKTI, 787 NFSV, 863 NISS, and 1196 NRTA) were absent from all reference strains. Further comparison showed that the 57 NSTW and 112 NATA sites in local field strains matched those in G2-lineage strains.

## 4. Discussion

Porcine epidemic diarrhea virus (PEDV) has undergone continuous genetic evolution since its emergence in China, resulting in recurrent outbreaks and substantial economic losses to the swine industry [20]. Despite widespread vaccination, field cases remain common, suggesting incomplete cross-protection between classical vaccine strains and currently circulating variants [21]. Sustained molecular surveillance is therefore essential to monitor viral evolution and guide evidence-based control strategies [22]. In the present study, PEDV was widely detected in diarrheic piglets across Chongqing, indicating active viral circulation in clinically affected herds. The overall positivity rate (48.31%) was comparable to levels reported in adjacent southwestern provinces, including Sichuan, Yunnan, and Guizhou, where prevalence has fluctuated across years but consistently reflected endemic rather than sporadic circulation [23,24,25]. These regional similarities support the notion that PEDV has transitioned from epidemic outbreaks to sustained endemic persistence in inland China, driven by continuous viral evolution and dense pig-production systems.

Phylogenetic analysis showed that all sequenced isolates belonged to the G2c lineage, consistent with recent reports describing the progressive dominance of this genotype nationally, including the gradual replacement of G2a and G2b variants after 2015 [22,26,27,28,29]. Similar genotype shifts have been documented in multi-provincial surveys, where G2c strains accounted for the majority of contemporary isolates, suggesting a stable evolutionary advantage linked to enhanced adaptability and immune selection pressure. The exclusive detection of G2c in this dataset was thus consistent with, rather than contradictory to, the broader national trend. Nevertheless, because only a limited, non-random subset of PEDV-positive samples was subjected to sequencing (18 samples were selected, and complete S gene sequences were successfully recovered from 15 isolates), the exclusive detection of G2c strains should be regarded as descriptive of the sampled dataset rather than conclusive evidence of population-level genotype predominance. Accordingly, the presence of less abundant lineages, such as G2a or G2b, cannot be excluded.

Genetic analysis of the S gene revealed moderate divergence from the classical CV777 strain, consistent with reports of rapid antigenic evolution in the spike protein as a key driver of immune escape and vaccine failure [30,31]. Conserved neutralizing epitope domains (SS2, SS6, 2C10) remained stable, while variability was concentrated within the COE region. Similar patterns have been reported in contemporary field strains, where mutations within the COE domain have been associated with altered antigenicity and reduced neutralization sensitivity [32]. Variation in the number and distribution of predicted N-glycosylation sites may further contribute to glycan shielding and reduced epitope accessibility, a mechanism also documented in studies of other PEDV strains and human/animal coronaviruses [33]. Together, these sequence- and structure-based observations suggest plausible mechanisms underlying antigenic drift among circulating Chongqing variants. However, as with comparable in silico analyses, these remain hypotheses: functional validation via virus-neutralization assays, antibody-binding studies, and reverse genetics will be needed to confirm whether these mutations and glycosylation changes meaningfully affect antigenicity, immune escape, or vaccine efficacy.

Recombination analysis further highlighted the important role of homologous recombination in PEDV evolution. Recombination is a well-recognized mechanism in coronaviruses as a driver of genomic diversification and fitness gain [34]. The recombination pattern identified here was broadly consistent with prior descriptions of G2c lineage formation, thought to have arisen from complex recombination between G2b and S-INDEL ancestors [35]. The relative uniformity of the recombination pattern among isolates in this study, however, suggested a regionally constrained event—potentially shaped by local immune and vaccination pressures—rather than ongoing, unstructured viral mixing.

Taken together, the findings supported a model in which contemporary PEDV evolution in Chongqing was driven by point mutation, homologous recombination, and glycosylation-mediated antigenic drift, consistent with national and regional trends. The persistence of PEDV despite vaccination likely reflected a broader interplay between viral evolution, pig-production density, and immune selection pressure. These findings underscore the importance of sustained molecular surveillance and expanded genomic sampling, as well as the integration of functional immunological assays to deepen our understanding of PEDV evolution and guide the development of next-generation vaccines targeting prevalent G2c variants.

Several limitations should be considered when interpreting the present findings. First, the study population consisted exclusively of diarrheic piglets and, therefore, the prevalence estimates were not representative of the general swine population, as PEDV prevalence and genetic characteristics may differ markedly in asymptomatic or subclinically infected animals. Consequently, the reported 48.31% positivity rate should not be extrapolated to the broader swine population and likely overestimates the true PEDV burden. Future surveillance efforts should incorporate systematic sampling across both symptomatic and asymptomatic cohorts to establish more robust epidemiological estimates. Second, genomic sequencing was performed on a limited, convenience-selected subset (*n* = 15 complete S gene sequences from 18 initially positive samples), which restricted our ability to comprehensively characterize the circulating genetic diversity. Third, conclusions regarding antigenicity and immune evasion were based primarily on sequence analyses, predicted glycosylation changes, and structural modeling. Although these approaches provided valuable insights into potential mechanisms of viral adaptation, they cannot substitute for functional validation. Future studies incorporating larger sample sizes, longitudinal surveillance, virus-neutralization assays, and antigenic profiling will be essential to clarify the biological significance of the observed mutations and their implications for vaccine performance.

## 5. Conclusions

This study provides molecular epidemiological evidence of PEDV circulation in diarrheic piglets in Chongqing between 2022 and 2024, with an RT-qPCR positivity rate of 48.31%. Phylogenetic analysis of representative isolates showed that all sequenced strains clustered within the G2c lineage, suggesting this lineage’s predominance in the Chongqing region during the study period. The detected viruses exhibited genetic divergence from the classical CV777 strain, including amino acid substitutions and variation in predicted N-glycosylation sites within antigenically important regions of the spike protein.

Although sequence-based recombination signals were identified among the analyzed isolates, and structural modeling suggested possible effects on antigenic regions, these findings should be interpreted cautiously as they are based on a limited number of sequences and in silico predictions without experimental validation. Therefore, the functional and epidemiological significance of these genetic changes remains to be confirmed.

Overall, the data highlight the ongoing genetic evolution of PEDV in Chongqing and underscore the importance of continuous molecular surveillance to monitor emerging variants. Larger-scale sequencing efforts combined with antigenic and functional studies are needed to better understand PEDV evolutionary dynamics and to support the development of effective and updated control strategies, including next-generation vaccines.

## Figures and Tables

**Table 1:** Potential N-Glycosylation sites on the S protein.

Legend	Strains	High-Specificity *N*-Glycosylation Sites of S Protein																							
		57 NSSS	112 +	127 NKTL	213 NVTS	230 NCTG	300 +	321 NDTS	341 NLSF	348 NSSD	378 NSTV	422 NFTG	511 NITV	553 NVTN	685 NVTS	740 NCTE	778 NISI	787 +	863 +	1006 NITS	1196 +	1229 NLTS	1246 NKTL	1258 NRTG	1275 NLTG
G1a	CV777	NSSS	-	NKTL	NVTS	NCTG	-	NDTS	NLSF	NSSD	NSTV	NFTG	NITV	NVTN	NVTS	NCTE	NISI	-	-	NITS	-	NLTS	NKTL	NRTG	NLTG
G1b	SD-M	NSSS	NTSA	NKTL	NVTS	-	-	-	NLSF	NSSD	-	NFTG	NITV	NVTN	NVTS	NCTE	NISI	-	-	NITS	-	NLTS	NKTL	-	NLTG
S-INDEL	OH851	NSSS	-	NKTL	NVTS	-	-	NDTS	NLSF	NSSD	NSTV	NFTG	NITV	NVTN	NVTS	NCTE	NISI	-	-	NISI	-	NLTR	NKTL	NRTG	NLTG
G2a	AH2012	NSTW	NATA	-	NVTS	-	-	NDTS	NFSF	NSSN	NSTV	NFTG	NITV	NVTN	NVTS	NCTE	NISI	-	-	NITS	-	NLTR	NKTL	NRTG	NLTG
G2b	AJ1102	NSTW	NATA	-	NVTS	-	-	NDTS	-	NSSD	NSTV	NFTG	NITV	NVTN	NVTS	NCTE	NISI	-	-	NITS	-	NLTR	NKTL	NRTG	NLTG
G2c	LYG	NSTW	NATA	-	NVTS	-	-	NDTS	NFSF	NSSN	NSTV	NFTG	NITV	NVTN	NVTS	NCTE	NISI	-	-	NITS	-	NLTR	NKTL	NRTG	NLTG
Endemic strain	CHN/SW33/2023	NSTW	NATA	-	NVTS	-	-	NDTS	NLSF	NSSN	NSTV	NFTG	NITV	NVTN	NVTS	NCTE	NISI	NFSV	-	NITS	-	NLTR	NKTL	NRTG	NLTG
Major parent	FR/001/2014	NSSS	-	NKTL	NVTS	-	-	NDTS	NLSF	NSSD	NSTV	NFTG	NITV	NVTN	NVTS	NCTE	NISI	-	-	NITS	-	NLTR	NKTL	NRTG	NLTG
Minor parent	CH/GDGZ/2012	NSTW	NATA	NKTL	NVTS	-	-	NDTS	-	NSSD	NSTV	NFTG	NITV	NVTN	NVTS	NCTE	NISI	-	-	NITS	-	NLTR	NKTL	NRTG	NLTG
This study	CQ/Fengdu/2023	NSTW	NATA	-	NVTS	-	NKTI	NDTS	-	NSSD	NSTV	NFTG	NITV	NVTN	NVTS	NCTE	NISI	-	NISS	NITS	-	NLTR	NKTL	NRTG	NLTG
This study	CQ/Dazu/2023	NSTW	NATA	-	NVTS	-	NKTI	NDTS	-	NSSD	NSTV	NFTG	NITV	NVTN	NVTS	NCTE	NISI	-	NISS	NITS	-	NLTR	NKTL	NRTG	NLTG
This study	CQ/Rongchang/2022	NSTW	NATA	-	NVTS	-	-	NGTS	NLSF	NSSD	NSTV	NFTG	NITV	NVTN	NVTS	NCTE	NISI	-	-	NITS	-	NLTR	NKTL	NRTG	NLTG
This study	CQ/Shizhu/2022	NSTW	NATA	-	NVTS	-	-	NDTS	NLSF	NSSD	NSTV	NFTG	NITV	NVTN	NVTS	NCTE	NISI	-	-	NITS	-	NLTR	NKTL	NRTG	NLTG
This study	CQ/Rongchang/2023	NSTW	NATA	-	NVTS	-	-	NDTS	NLSF	NSSD	NSTV	NFTG	NITV	NVTN	NVTS	NCTE	NISI	-	-	NITS	-	NLTR	NKTL	NRTG	NLTG
This study	CQ/Pengshui/2023	NSTW	NATA	-	NVTS	-	-	NDTS	NLSF	NSSD	NSTV	NFTG	NITV	NVTN	NVTS	NCTE	NISI	-	-	NITS	-	NLTR	NKTL	NRTG	NLTG
This study	CQ/Wulong/2023	NSTW	NATA	-	NVTS	-	-	NDTS	NLSF	NSSD	NSTV	NFTG	NITV	NVTN	NVTS	NCTE	NISI	-	-	NITS	NRTA	NLTR	NKTL	NRTG	NLTG
This study	CQ/Yongchuan/2023	NSTW	NATA	-	NVTS	-	-	NDTS	NLSF	NSSN	NSTV	NFTG	NITV	NVTN	NVTS	NCTE	NISI	NFSV	-	NITS	NRTA	NLTR	NKTL	NRTG	NLTG
This study	CQ/Changshou/2024	NSTW	NATA	-	NVTS	-	-	NDTS	NLSF	NSSD	NSTV	NFTG	NITV	NVTN	NVTS	NCTE	NISI	-	-	NITS	-	NLTR	NKTL	NRTG	NLTG
This study	CQ/Hechuan/2024	NSTW	NATA	-	NVTS	-	-	NDTS	NLSF	NSSN	NSTV	NFTG	NITV	NVTN	NVTS	NCTE	NISI	-	-	NITS	-	NLTR	NKTL	NRTG	NLTG
This study	CQ/Fengdu/2022	NSTW	NATA	-	NVTS	-	-	NDTS	NLSF	NSSD	NSTV	NFTG	NITV	NVTN	NVTS	NCTE	NISI	-	-	NITS	-	NLTR	NKTL	NRTG	NLTG
This study	CQ/Hechuan/2022	NSTW	NATA	-	NVTS	-	-	NDTS	NLSF	NSSN	NSTV	NFTG	NITV	NVTN	NVTS	NCTE	NISI	-	-	NITS	-	NLTR	NKTL	NRTG	NLTG
This study	CQ/Yongchuan/2024	NSTW	NATA	-	NVTS	-	-	NDTS	NLSF	NSSN	NSTV	NFTG	NITV	NVTN	NVTS	NCTE	NISI	NFSV	-	NITS	-	NLTR	NKTL	NRTG	NLTG
This study	CQ/Shizhu/2024	NSTW	NATA	-	NVTS	-	-	NDTS	NLSF	NSSN	NSTV	NFTG	NITV	NVTN	NVTS	NCTE	NISI	-	-	NITS	-	NLTR	NKTL	NRTG	NLTG
This study	CQ/Wulong/2022	NSTW	NATA	-	NVTS	-	-	NDTS	NLSF	NSSN	NSTV	NFTG	NITV	NVTN	NVTS	NCTE	NISI	NFSV	-	NITS	-	NLTR	NKTL	NRTG	NLTG

Note: Compared with the prototype strain CV777, “+”: Newly identified site; “-”: Absent site. Color key (vs. CV777): conserved N-glycosylation sites (green); mutated or lineage-specific sites (yellow); novel sites identified (blue).

## Data Availability

The original contributions presented in this study are included in the article/Supplementary Materials. Further inquiries can be directed to the corresponding authors.

## Supplementary Materials

The following supporting information can be downloaded at https://www.mdpi.com/article/10.3390/ani16132033/s1.

## References

[1] Jung K., Saif L.J., Wang Q. (2020). Porcine epidemic diarrhea virus (PEDV): An update on etiology, transmission, pathogenesis, and prevention and control. Virus Res..

[2] Wang Q., Vlasova A.N., Kenney S.P., Saif L.J. (2019). Emerging and re-emerging coronaviruses in pigs. Curr. Opin. Virol..

[3] Pensaert M.B., de Bouck P. (1978). A new coronavirus-like particle associated with diarrhea in swine. Arch. Virol..

[4] Xuan H., Xing D., Wang D., Zhu W., Zhao F., Gong H., Fei S. (1984). Study on the culture of porcine epidemic diarrhea virus adapted to fetal porcine intestine primary cell monolayer. Chin. J. Vet. Sci..

[5] Wang J., Zhao P., Guo L., Liu Y., Du Y., Ren S., Li J., Zhang Y., Fan Y., Huang B. (2013). Porcine epidemic diarrhea virus variants with high pathogenicity, China. Emerg. Infect. Dis..

[6] Cima G. (2014). PED virus reinfecting U.S. herds. Virus estimated to have killed 7 million-plus pigs. J. Am. Vet. Med. Assoc..

[7] Lee C. (2015). Porcine epidemic diarrhea virus: An emerging and re-emerging epizootic swine virus. Virol. J..

[8] Ojkic D., Hazlett M., Fairles J., Marom A., Slavic D., Maxie G., Alexandersen S., Pasick J., Alsop J., Burlatschenko S. (2015). The first case of porcine epidemic diarrhea in Canada. Can. Vet. J..

[9] Chambers P., Pringle C.R., Easton A.J. (1990). Heptad repeat sequences are located adjacent to hydrophobic regions in several types of virus fusion glycoproteins. J. Gen. Virol..

[10] Stott C.J., Temeeyasen G., Tripipat T., Kaewprommal P., Tantituvanont A., Piriyapongsa J., Nilubol D. (2017). Evolutionary and epidemiological analyses based on spike genes of porcine epidemic diarrhea virus circulating in Thailand in 2008–2015. Infect. Genet. Evol..

[11] Zhao Y., Fan B., Song X., Gao J., Guo R., Yi C., He Z., Hu H., Jiang J., Zhao L. (2024). PEDV-spike-protein-expressing mRNA vaccine protects piglets against PEDV challenge. mBio.

[12] Huang Y., Dickerman A.W., Piñeyro P., Li L., Fang L., Kiehne R., Opriessnig T., Meng X. (2013). Origin, evolution, and genotyping of emergent porcine epidemic diarrhea virus strains in the United States. mBio.

[13] Su M., Li C., Qi S., Yang D., Jiang N., Yin B., Guo D., Kong F., Yuan D., Feng L. (2020). A molecular epidemiological investigation of PEDV in China: Characterization of co-infection and genetic diversity of S1-based genes. Transbound. Emerg. Dis..

[14] Zhang Y., Chen Y., Zhou J., Wang X., Ma L., Li J., Yang L., Yuan H., Pang D., Ouyang H. (2022). Porcine Epidemic Diarrhea Virus: An Updated Overview of Virus Epidemiology, Virulence Variation Patterns and Virus–Host Interactions. Viruses.

[15] Sun J., Cheng J., Shi D., Xu X., Liu Y., Ying J., Zhao Y., Zheng H., Yan J., Sun D. (2025). Genetic Epidemiology of Porcine Epidemic Diarrhea Virus Circulating in China From 2010 to 2024: Characterization of Phylogenetic and Genetic Diversity of S1-Based Genes. J. Med. Virol..

[16] Zhang H., Zou C., Peng O., Ashraf U., Xu Q., Gong L., Fan B., Zhang Y., Xu Z., Xue C. (2023). Global Dynamics of Porcine Enteric Coronavirus PEDV Epidemiology, Evolution, and Transmission. Mol. Biol. Evol..

[17] Fu Y., Wang Y., Dai L., Cheng B., Xiao S., Yin Y. (2025). Evolutionary dynamics and antigenic diversity of porcine epidemic diarrhea virus (PEDV) in China: Phylogenetic and recombination analyses based on large-scale S gene sequences. BMC Vet. Res..

[18] Chen Q., Li S., Zhang Y., Mu H., Liu M., Yang L., Guo Q., Fu L. (2025). Development of a triplex quantitative reverse transcription-polymerase chain reaction for the detection of porcine epidemic diarrhea virus, transmissible gastroenteritis virus, and porcine delta coronavirus. Chin. J. Vet. Sci..

[19] Chang S.-H., Bae J.-L., Kang T.-J., Kim J., Chung G.-H., Lim C.-W., Laude H., Yang M.-S., Jang Y.-S. (2002). Identification of the epitope region capable of inducing neutralizing antibodies against the porcine epidemic diarrhea virus. Mol. Cells.

[20] Sun R., Cai R., Chen Y., Liang P., Chen D., Song C. (2012). Outbreak of porcine epidemic diarrhea in suckling piglets, China. Emerg. Infect. Dis..

[21] Park J.-E. (2024). Porcine Epidemic Diarrhea: Insights and Progress on Vaccines. Vaccines.

[22] Gao M., Liu Y., Xu X., Chen P., Gong L., Wang H., Sun Y., Chen H. (2026). Temporal evolutionary dynamics of porcine epidemic diarrhea virus in China from 2013 to 2023. Infect. Genet. Evol..

[23] Xiao L., Kang R., Wu X., Yu J., Yang J., Mao C., Xie J., Ye Y., Li X., Wei Y. (2026). Prevalence and S gene characterization of porcine epidemic diarrhea virus in Sichuan province, China (2023–2024). Front. Vet. Sci..

[24] Zhu P., Yuan H., Shu X., Li X., Cui Y., Gao L., Yan R., Yu T., Song C., Yao J. (2025). Epidemiological Study and Genetic Diversity Assessment of Porcine Epidemic Diarrhea Virus (PEDV) in Yunnan Province, China. Viruses.

[25] Tian H., Liu J., Liang H., Tang D., Wang B., Bian M., Huang S. (2024). Epidemiological investigation on the prevalence of PEDV and PoRVA in Guizhou during 2017–2023 and genetic evolution analysis of VP7 and VP4 genes of PoRVA strains. Chin. J. Prev. Vet. Med..

[26] Peng Q., Fu P., Zhou Y., Lang Y., Zhao S., Wen Y., Wang Y., Wu R., Zhao Q., Du S. (2024). Phylogenetic Analysis of Porcine Epidemic Diarrhea Virus (PEDV) during 2020–2022 and Isolation of a Variant Recombinant PEDV Strain. Int. J. Mol. Sci..

[27] Li Y., Fan C., Li H., Zhen H., Zhu Y., Wang S., Wang B., Huang Y. (2026). Emergence of a Highly Virulent Porcine Epidemic Diarrhea Virus (PEDV) G2c Subtype in China: Isolation, Genetic and Pathogenic Characterization, and Cross-Neutralizing Antibody Response. Transbound. Emerg. Dis..

[28] Xu H., Wu H., Min J., Fu N., Shi Y., Zhou P., Wang M., Shi A., Zhou Y., Chen J. (2025). Effective control of the emerging PEDV G2-c variant with an inactivated autogenous vaccine. Front. Vet. Sci..

[29] Lu X., Chen C., Wang Z., Zhang A. (2025). Isolation and Characterization of Porcine Epidemic Diarrhea Virus G2c Strains Circulating in China from 2021 to 2024. Vet. Sci..

[30] Zhuang H., Sun L., Wang X., Xiao M., Zeng L., Wang H., Yang H., Lin F., Wang C., Qin L. (2022). Molecular characterization and phylogenetic analysis of porcine epidemic diarrhea virus strains circulating in China from 2020 to 2021. BMC Vet. Res..

[31] Luo H., Liang Z., Lin J., Wang Y., Liu Y., Mei K., Zhao M., Huang S. (2024). Research progress of porcine epidemic diarrhea virus S protein. Front. Microbiol..

[32] Mannar D., Saville J.W., Sun Z., Zhu X., Marti M.M., Srivastava S.S., Berezuk A.M., Zhou S., Tuttle K.S., Sobolewski M.D. (2022). SARS-CoV-2 variants of concern: Spike protein mutational analysis and epitope for broad neutralization. Nat. Commun..

[33] Wang Z., Yang K., Bi M., Li K., Wang W., Song Y., Pan X., Li T., Mo X. (2025). Molecular characteristics and potential antigenic epitope analysis of porcine epidemic diarrhea virus in China from 2022 to 2025. Front. Vet. Sci..

[34] Liu C., Tang J., Ma Y., Liang X., Yang Y., Peng G., Qi Q., Jiang S., Li J., Du L. (2015). Receptor usage and cell entry of porcine epidemic diarrhea coronavirus. J. Virol..

[35] Li X., Li Y., Huang J., Yao Y., Zhao W., Zhang Y., Qing J., Ren J., Yan Z., Wang Z. (2022). Isolation and oral immunogenicity assessment of porcine epidemic diarrhea virus NH-TA2020 strain: One of the predominant strains circulating in China from 2017 to 2021. Virol. Sin..

