# Supplementary material for: Genetic Diversity and Molecular Evolution of Porcine Epidemic Diarrhea Virus in Chongqing, China (2022–2024)
Source: Animals (Basel). 2026 Jul 2;16(13):2033. doi: 10.3390/ani16132033 (PMC13359853; doi:10.3390/ani16132033)
Supplement: Supplementary file 1 [file animals-16-02033-s001.zip › animals-4363302-supplementary.pdf]

Table S1. Sample information and positive rate

Region	District	Total samples	Total positives	Positivity rate (%)	2022			2023			2024			Total samples	Total positives	Positivity rate (%)
					Samples	Positives	Rate (%)	Samples	Positives	Rate (%)	Samples	Positives	Rate (%)			
Core urban area	Yongchuan	42	26	61.90%	4	1	25.00%	27	19	70.37%	11	6	54.55%	174	98	56.32%
	Hechuan	29	16	55.17%	/	/	/	22	13	59.09%	7	3	42.86%			
	Rongchang	37	17	45.95%	9	3	33.33%	16	11	68.75%	12	3	25.00%			
	Dazu	30	19	63.33%	/	/	/	14	7	50.00%	16	12	75.00%			
	Changshou	36	20	55.56%	10	4	40.00%	15	8	53.33%	11	8	72.73%			
NE Chongqing Three Gorges Reservoir	Fengdu	33	12	36.36%	/	/	/	13	4	30.77%	20	8	40.00%	33	12	36.36%
SE Chongqing Wuling Mountains	Shizhu	24	14	58.33%	6	2	33.33%	11	6	54.55%	7	6	85.71%	89	33	37.08%
	Wulong	37	9	24.32%	/	/	/	/	/	/	37	9	24.32%			
	Pengshui	28	10	35.71%	6	1	16.67%	10	4	40.00%	12	5	41.67%			
Total		296	143	48.31%	/											

Table S2. Primers and probes

Group	Primer/Probe	Sequence（5′ to 3′）	Amplicon length (bp)	Accession no.
RT-qPCR	PEDV M128F	GTTGCTACTGGCGTACAGGT	128	KX852340.1
	PEDV M128R	TAGAAAGCCCAACCAGTGC		
	PEDV M128P	FAM-TCGTCACAGTCGCCAAGGCCA-BHQ1		
S1	PEDV-S1F	ATGAAGTCTTTAACTTACTTCTGGTTGTT	2376	OR085231.1
	PEDV-S1R	AATACTCATACTAAAGTTGGTGGGA		
S2	PEDV-S2F	ACAGAGCCTGTGTTGGTGTAT	1926	
	PEDV-S2R	TCACTGCACGTGGACCTTTT		

Table S3. Sequence information

No.	Virus strain	Accession no.	Time	Laction	Source
1	CV777	AF353511.1	1978	Belgium	GenBank
2	SM98	GU937797.1	1998	Korea	GenBank
3	LZC	EF185992.1	2006	China	GenBank
4	CHM2013	KM887144.1	2013	China	GenBank
5	DR13	JQ023161.1	1999	Korea	GenBank
6	AH-M	KJ158152.1	2011	China	GenBank
7	SD-M	JX560761.1	2012	China	GenBank
8	JS2008	KC109141.1	2008	China	GenBank
9	Attenuated DR13	JQ023162.1	2011	Korea	GenBank
10	SQ2014	KP728470.1	2014	China	GenBank
11	PEDV-SX	KY420075.1	2015	China	GenBank
12	OH851	KJ399978.1	2013	USA	GenBank
13	FR/001/2014	KR011756.1	2014	France	GenBank
14	USA/Iowa106/2013	KJ645695.1	2013	USA	GenBank
15	USA/Ohio126/2014	KJ645702.1	2014	USA	GenBank
16	MYZ-1/JPN/2013	LC063846.1	2013	Japan	GenBank
17	ZL29	KU847996.1	2015	China	GenBank
18	GER/L00719/2014	LM645058.1	2014	Germany	GenBank
19	AH2012	KC210145.1	2012	China	GenBank
20	BJ-2011-1	JN825712.1	2011	China	GenBank
21	CH/ZMDZY/11	KC196276.1	2011	China	GenBank
22	JS-HZ2012	KC210147.1	2012	China	GenBank
23	GD-B	JX088695.1	2012	China	GenBank
24	USA/Colorado/2013	KF272920.1	2013	USA	GenBank
25	ISU13-22038-IA	KF650373.1	2013	USA	GenBank
26	IA2	KF468754.1	2013	USA	GenBank
27	MN	KF468752.1	2013	USA	GenBank
28	GD-A	JX112709.1	2012	China	GenBank
29	CH/FJND-3/2011	JQ282909.1	2011	China	GenBank
30	CH/FJZZ-9/2012	KC140102.1	2012	China	GenBank
31	CH/GDGZ/2012	KF384500.1	2012	China	GenBank
32	AJ1102	JX188454.1	2011	China	GenBank
33	LC	JX489155.1	2011	China	GenBank
34	GD-1	JX647847.1	2011	China	GenBank
35	CH/HLJJS/2022	ON968723.1	2022	China	GenBank
36	AHCZ02	PQ682538.1	2022	China	GenBank
37	2024-JXYX	PV235414.1	2024	China	GenBank
38	2024-JXNC	PV235413.1	2024	China	GenBank
39	2021-HBMC	PV235415.1	2021	China	GenBank
40	GNU-2120	ON263454.1	2021	South Korea	GenBank
41	JS-2023	PX733332.1	2023	China	GenBank
42	KP2022-1	OR125555.1	2022	South Korea	GenBank
43	CH/SCCD/12/2020	MZ161009.1	2020	China	GenBank
44	CH/GZZY/12/2020	MZ161063.1	2020	China	GenBank
45	GD-GZ2020-9	PP580826.1	2020	China	GenBank
46	JLBC9-2023	PV536097.1	2023	China	GenBank
47	CH/SCCZ/2017	MH053419.1	2017	China	GenBank
48	CHN/SW29/2022	PV146390.1	2022	China	GenBank
49	CH-SCCQ-2019	MK820041.1	2019	China	GenBank
50	GZFQ/2021	OQ122104.1	2021	China	GenBank
51	CH-SCYA-2019	MK820040.1	2019	China	GenBank

52	CH/HNJZ-01/2020	MZ570138.1	2020	China	GenBank
53	CH/SCQL-1/2018	MN617866.1	2018	China	GenBank
54	JSYC/2021	OQ122084.1	2021	China	GenBank
55	CH/SCYBZH/12/2020	MZ161003.1	2020	China	GenBank
56	CH/Sichuan-T/2022	PP723330.1	2022	China	GenBank
57	CN/Hainan/Sinder202112-4	ON248877.1	2021	China	GenBank
58	CH/HN/NK1/2021	OP019340.1	2021	China	GenBank
59	CHN/Hainan/Sinder202205-1	OP374016.1	2022	China	GenBank
60	CHN/SW30/2023	PV146391.1	2023	China	GenBank
61	CH-HK-2021	PP785988.1	2021	China	GenBank
62	CH/MM1/FS-12/2021	OM974578.1	2021	China	GenBank
63	SD2021	OL762459.1	2021	China	GenBank
64	CHN/SW32/2023	PV146393.1	2023	China	GenBank
65	ZJ23HZ0201	PV164724.1	2023	China	GenBank
66	MY2404	PV641628.1	2024	China	GenBank
67	AH06-04-CN-2025	PX769334.1	2025	China	GenBank
68	SC2022GY	PP395591.1	2022	China	GenBank
69	JS-SQMV202102	PX229697.1	2021	China	GenBank
70	AH03-11-CN-2021	PV523963.1	2024	China	GenBank
71	CHhbzd2025	PX777773.1	2025	China	GenBank
72	MS2302	PV614327.1	2023	China	GenBank
73	G2c	PV844376.1	2024	China	GenBank
74	CH/SC/CY/2022	OP019350.1	2022	China	GenBank
75	CD2302	PV609793.1	2023	China	GenBank
76	CH/ShXXY2-2023	PQ316092.1	2023	China	GenBank
77	CH-HeN24-2023	OQ718904.1	2023	China	GenBank
78	HN24HN0401	PV164731.1	2024	China	GenBank
79	AH02-02-CN-2025	PX769328.1	2025	China	GenBank
80	CHsxzd2025	PX777772.1	2025	China	GenBank
81	AH06-11-CN-2024	PV523969.1	2024	China	GenBank
82	CHN/SW33/2023	PV146394.1	2023	China	GenBank
83	JS01-01-CN-2023	PV523984.1	2023	China	GenBank
84	CH-HeN31-2023	OQ718910.1	2023	China	GenBank
85	SC/MY/2023	PQ507870.1	2023	China	GenBank
86	CH-HeN25-2023	OQ718905.1	2023	China	GenBank
87	SC2020NC	PP395573.1	2020	China	GenBank
88	LYG	KM609212.1	2014	China	GenBank
89	CQ/Fengdu/2023	PZ542161	2023	China/Chongqing	This study
90	CQ/Dazu/2023	PZ542160	2023	China/Chongqing	This study
91	CQ/Rongchang/2022	PZ542159	2022	China/Chongqing	This study
92	CQ/Shizhu/2022	PZ542158	2022	China/Chongqing	This study
93	CQ/Rongchang/2023	PZ542157	2023	China/Chongqing	This study
94	CQ/Pengshui/2023	PZ542156	2023	China/Chongqing	This study
95	CQ/Wulong/2023	PZ542155	2023	China/Chongqing	This study
96	CQ/Yongchuan/2023	PZ542154	2023	China/Chongqing	This study
97	CQ/Changshou/2024	PZ542153	2024	China/Chongqing	This study
98	CQ/Hechuan/2024	PZ542152	2024	China/Chongqing	This study
99	CQ/Fengdu/2022	PZ542151	2022	China/Chongqing	This study
100	CQ/Hechuan/2022	PZ542150	2022	China/Chongqing	This study
101	CQ/Yongchuan/2024	PZ542149	2024	China/Chongqing	This study
102	CQ/Shizhu/2024	PZ542148	2024	China/Chongqing	This study
103	CQ/Wulong/2022	PZ545697	2022	China/Chongqing	This study

Table S4. Nucleotide identity

	CV777-AF353	SD-M-JX5607	OH851-KJ399	AH2012-KC21	AJ1102-JX18	LYG-KM6092	CHN/SW33/20	CQ-Fengdu-20	CQ-Dazu-202	CQ-Rongchang	CQ-Shizhu-20	CQ-Rongchang	CQ-Pengshui-2	CQ-Wulong-20	CQ-Yongchuan	CQ-Changshou	CQ-Hechuan-2	CQ-Fengdu-20	CQ-Hechuan-2	CQ-Yongchuan	CQ-Shizhu-20	CQ-Wulong-2022
CV777-AF353511.1																						
SD-M-JX560761.1	96.84																					
OH851-KJ399978.1	95.79	95.90																				
AH2012-KC210145.1	94.28	94.13	96.14																			
AJ1102-JX188454.1	94.33	93.96	95.24	97.96																		
LYG-KM609212.1	94.21	94.04	96.65	98.61	97.57																	
CHN/SW33/2023-PV146394.1	94.09	93.94	96.29	98.46	97.45	98.87																
CQ-Fengdu-2023	93.48	93.29	95.44	97.35	97.04	98.10	97.74															
CQ-Dazu-2023	93.48	93.29	95.44	97.35	97.04	98.10	97.74	99.95														
CQ-Rongchang-2022	93.37	93.13	95.48	97.49	96.79	98.07	98.00	97.57	97.57													
CQ-Shizhu-2022	93.59	93.35	95.84	97.90	96.91	98.43	98.41	97.45	97.45	99.54												
CQ-Rongchang-2023	94.04	93.77	96.14	98.22	97.40	98.77	98.85	97.74	97.74	97.90	98.31											
CQ-Pengshui-2023	93.97	93.70	96.07	98.15	97.33	98.70	98.77	97.67	97.67	97.83	98.24	99.93										
CQ-Wulong-2023	93.87	93.58	95.95	98.03	97.21	98.58	98.65	97.57	97.57	97.76	98.17	99.81	99.74									
CQ-Yongchuan-2023	93.99	93.87	96.24	98.41	97.40	98.82	99.76	97.74	97.74	97.90	98.31	98.99	98.92	98.85								
CQ-Changshou-2024	94.07	93.85	96.21	98.29	97.47	98.85	99.11	97.81	97.81	98.02	98.43	99.74	99.66	99.54	99.01							
CQ-Hechuan-2024	94.02	93.92	96.26	98.44	97.43	98.85	99.88	97.72	97.72	97.98	98.39	98.82	98.75	98.63	99.69	99.09						
CQ-Fengdu-2022	93.87	93.74	96.26	98.29	97.30	98.87	98.85	97.79	97.79	99.01	99.42	98.75	98.68	98.56	98.75	98.87	98.82					
CQ-Hechuan-2022	93.82	93.62	95.73	97.69	97.67	98.65	98.15	98.10	98.10	97.64	97.66	98.00	97.93	97.81	98.12	98.12	98.12	98.10				
CQ-Yongchuan-2024	94.09	93.97	96.33	98.51	97.50	98.92	99.90	97.79	97.79	98.05	98.46	98.89	98.82	98.70	99.86	99.16	99.83	98.89	98.22			
CQ-Shizhu-2024	93.92	93.85	96.16	98.34	97.33	98.75	99.69	97.62	97.62	97.88	98.29	98.73	98.65	98.53	99.59	98.99	99.66	98.73	98.03	99.74		
CQ-Wulong-2022	93.80	93.70	96.07	98.25	97.16	98.68	99.09	97.62	97.62	98.53	98.94	98.56	98.49	98.41	98.99	98.82	99.01	99.09	98.05	99.13	98.97	
MAX	94.09	93.97	96.33	98.51	97.67	98.92	99.90	99.95														
Min	93.37	93.13	95.44	97.35	96.79	98.07	97.74	97.45														

Table S5. Amino acid identity

	CV777-AF3535	SD-M-JX56076	OH851-KJ3999	AH2012-KC21(	AJ1102-JX188	LYG-KM60921	CHN/SW33/202	CQ/Fengdu/202	CQ/Dazu/2023	CQ/Rongchang/	CQ/Shizhu/2022	CQ/Rongchang/	CQ/Pengshui/20	CQ/Wulong/202	CQ/Yongchuan/	CQ/Changshou/	CQ/Hechuan/20	CQ/Fengdu/202	CQ/Hechuan/20	CQ/Yongchuan/	CQ/Shizhu/2024	CQ/Wulong/2022
CV777-AF353511.1																						
SD-M-JX560761.1	95.80																					
OH851-KJ399978.1	96.17	95.80																				
AH2012-KC210145.1	93.56	92.68	95.73																			
AJ1102-JX188454.1	93.70	93.04	95.36	98.19																		
LYG-KM609212.1	93.56	92.97	96.02	98.63	98.12																	
CHN/SW33/2023-PV146394.1	93.48	92.75	95.80	98.34	97.83	98.85																
CQ/Fengdu/2023	93.04	92.39	94.42	96.97	97.33	97.33	97.18															
CQ/Dazu/2023	93.04	92.39	94.42	96.97	97.33	97.33	97.18	99.86														
CQ/Rongchang/2022	92.88	92.08	94.63	97.18	97.25	97.47	97.61	96.67	96.67													
CQ/Shizhu/2022	93.03	92.23	95.07	97.69	97.32	97.98	98.12	96.60	96.60	99.49												
CQ/Rongchang/2023	93.56	92.83	95.87	98.27	98.05	98.77	98.77	97.11	97.11	97.54	98.05											
CQ/Pengshui/2023	93.34	92.61	95.66	98.05	97.83	98.56	98.56	96.90	96.90	97.32	97.83	99.78										
CQ/Wulong/2023	93.27	92.46	95.44	97.84	97.69	98.34	98.34	96.82	96.82	97.32	97.76	99.57	99.35									
CQ/Yongchuan/2023	93.34	92.75	95.73	98.27	97.83	98.77	99.64	97.18	97.18	97.61	98.05	98.85	98.63	98.56								
CQ/Changshou/2024	93.48	92.75	95.80	98.20	97.98	98.70	98.99	97.18	97.18	97.47	97.98	99.78	99.57	99.35	98.92							
CQ/Hechuan/2024	93.27	92.68	95.73	98.27	97.76	98.77	99.64	97.11	97.11	97.54	98.05	98.70	98.48	98.27	99.42	98.92						
CQ/Fengdu/2022	93.41	92.82	95.87	98.34	97.98	98.84	98.99	97.18	97.18	98.34	98.84	98.92	98.70	98.48	98.92	98.84	98.92					
CQ/Hechuan/2022	93.12	92.46	94.93	97.55	98.05	98.19	98.05	97.76	97.76	97.32	97.25	97.98	97.76	97.69	98.12	97.91	97.98	98.05				
CQ/Yongchuan/2024	93.41	92.83	95.87	98.41	97.91	98.92	99.78	97.26	97.26	97.68	98.19	98.85	98.63	98.41	99.86	99.06	99.57	99.06	98.19			
CQ/Shizhu/2024	93.27	92.68	95.73	98.27	97.76	98.77	99.49	97.11	97.11	97.54	98.05	98.70	98.48	98.27	99.42	98.92	99.42	98.92	97.98	99.57		
CQ/Wulong/2022	92.90	92.25	95.15	97.91	97.26	98.27	98.99	96.82	96.82	97.83	98.34	98.20	97.98	97.91	98.92	98.41	98.77	98.56	97.83	99.06	98.92	
Min	92.88	92.08	94.42	96.97	97.25	97.33	97.18	96.60														
MAX	93.56	92.83	95.87	98.41	98.05	98.92	99.78	99.86														
